# Genetic differentiation of the pine processionary moth at the southern edge of its range: contrasting patterns between mitochondrial and nuclear markers

**DOI:** 10.1002/ece3.2194

**Published:** 2016-05-26

**Authors:** M'hamed El Mokhefi, Carole Kerdelhué, Christian Burban, Andrea Battisti, Gahdab Chakali, Mauro Simonato

**Affiliations:** ^1^ Département de Zoologie Agricole et ForestièreEcole Nationale Supérieure AgronomiqueEl‐Harrach16200AlgiersAlgeria; ^2^ INRAUMR CBGPF‐34988Montferrier‐sur‐LezFrance; ^3^ BIOGECOINRA, Univ. Bordeaux33610CestasFrance; ^4^ Department of Agronomy, Food, Natural Resources, Animals, & Environment (DAFNAE)University of PaduaViale dell'Università 1635020Legnaro (PD)Italy

**Keywords:** Algeria, *Cedrus*, insects, Maghreb, phylogeography, *Pinus*

## Abstract

The pine processionary moth (*Thaumetopoea pityocampa*) is an important pest of coniferous forests at the southern edge of its range in Maghreb. Based on mitochondrial markers, a strong genetic differentiation was previously found in this species between western (*pityocampa* clade) and eastern Maghreb populations (ENA clade), with the contact zone between the clades located in Algeria. We focused on the moth range in Algeria, using both mitochondrial (a 648 bp fragment of the tRNA‐cox2) and nuclear (11 microsatellite loci) markers. A further analysis using a shorter mtDNA fragment and the same microsatellite loci was carried out on a transect in the contact zone between the mitochondrial clades. Mitochondrial diversity showed a strong geographical structure and a well‐defined contact zone between the two clades. In particular, in the *pityocampa* clade, two inner subclades were found whereas ENA did not show any further structure. Microsatellite analysis outlined a different pattern of differentiation, with two main groups not overlapping with the mitochondrial clades. The inconsistency between mitochondrial and nuclear markers is probably explained by sex‐biased dispersal and recent afforestation efforts that have bridged isolated populations.

## Introduction

Determining species boundaries in a set of closely related species is essential for biodiversity and evolutionary studies as well as for pest management and other areas of applied biology. This task is often complicated by the presence of cryptic species, morphologically not distinguishable (Bickford et al. 2007) although differing in biologically important traits such as host specificity, phenology, and susceptibility to natural enemies (Hebert et al. 2004; Garros et al. 2006; de Leon and Nadler 2010). Molecular phylogenetic evidence can provide useful information to sort out new taxa among cryptic species (Brower 1996; Pons et al. 2006). The use of maternally inherited mitochondrial genes is useful in this concern although it may prevent a correct classification due to potential introgression, hybridization, and incomplete lineage sorting (Valentini et al. 2009). Thus, empirical evidence of species delimitation should come from concordant genetic partitions across multiple and independent molecular markers.

**Figure 1:**
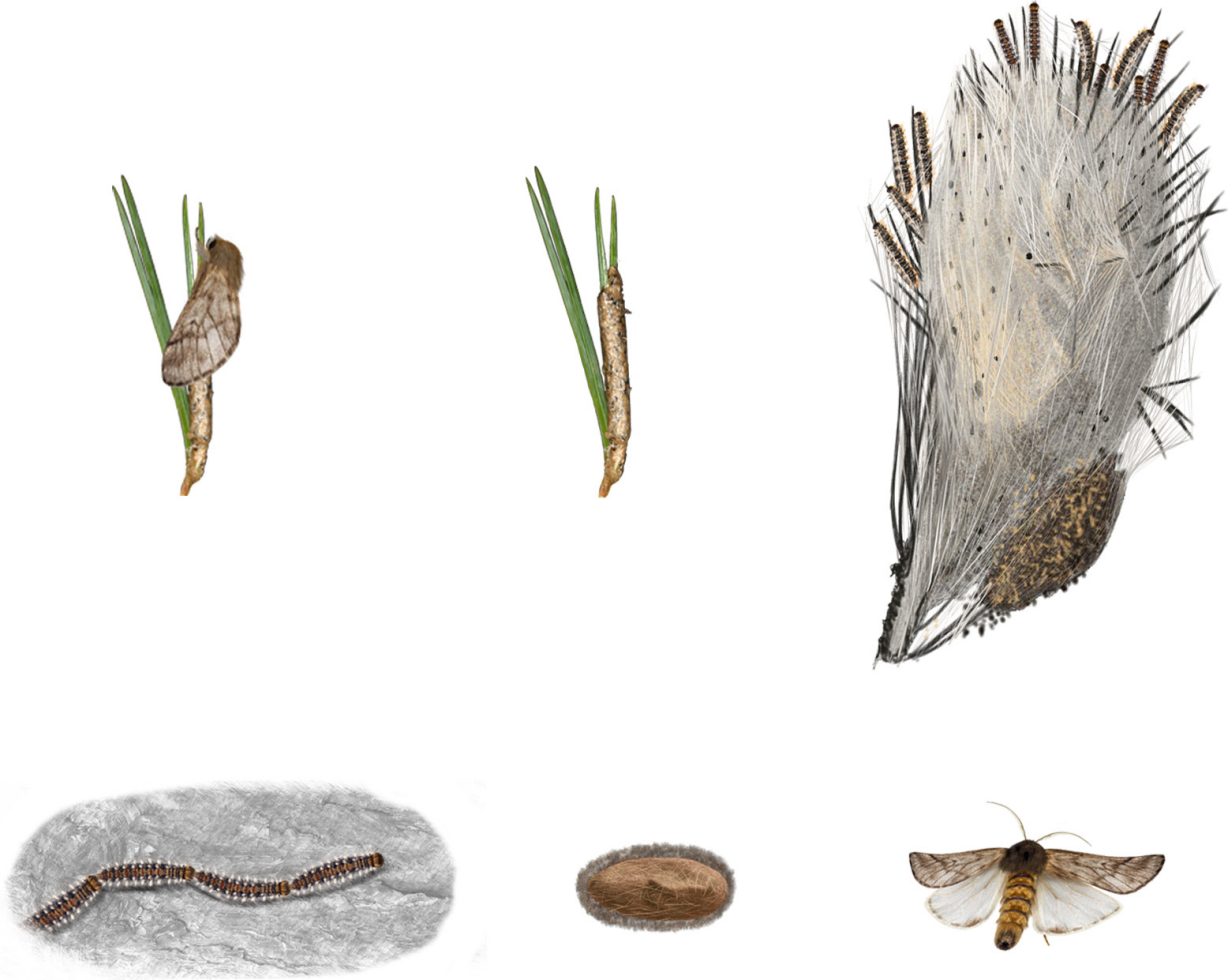
Pictorial sketch of the life history of the pine processionary moth *Thaumetopoea pityocampa*. The moths lay their eggs in a batch on the needles during the summer and the larvae spin a silky tent where they spend a long period across the winter. They rest inside during the day and forage during the night. In spring, they leave the tent and form long processions to the ground, where they pupate inside a cocoon about 10 cm deep. The moths emerge from the soil during summer (drawing of Paolo Paolucci).

The winter pine processionary moth is one of the main forest pests in the Mediterranean countries, with larvae feeding across the winter, and adults spreading and reproducing in the summer, after a pupation period in the soil lasting from spring to summer, or for one or more years of prolonged diapause (Battisti et al. 2015; Fig. 1). Larvae are gregarious and develop in a typical silk tent, with a defense system based on urticating setae, which are also harmful to humans and domestic animals (Battisti et al. 2011). The winter pine processionary moth is a complex of two closely related and morphologically very similar species, *Thaumetopoea pityocampa* (Denis and Schiffermüller), and *Thaumetopoea wilkinsoni* (Tams), with *T. pityocampa* found in southern Europe and northern Africa, and *T. wilkinsoni* in the Near East. They diverged at the end of the Miocene (7.5 Mya; Kerdelhué et al. 2009). Both species show a strong phylogeographic structure (Salvato et al. 2002; Simonato et al. 2007), which seems to be linked to the limited dispersal of female moths (Battisti et al. 2015) and to past climatic events (Kerdelhué et al. 2015).

A further and unexpected genetic structure was found inside *T. pityocampa*, where two very distinct mitochondrial clades were identified, almost as old as the *T. pityocampa/T. wilkinsoni* subdivision (6.7 Mya) (Kerdelhué et al. 2009). The “*pityocampa* clade” sensu stricto was found to occur in Europe and western Maghreb (Morocco and south‐western Algeria) whereas the eastern Maghreb populations (“ENA clade”) were limited to eastern North Africa (eastern Algeria, Tunisia, and Libya) (Kerdelhué et al. 2009). The likely geographical limit between the two mitochondrial clades was identified in Algeria (Kerdelhué et al. 2009), but the possible existence of a contact zone was not addressed. Moreover, the nuclear differentiation among moth populations in this region was not considered.

To fill this gap, we conducted a large survey of pine processionary moth populations in Algeria using mitochondrial and nuclear DNA markers. The sampling scheme included the most common native hosts, such as the ubiquitous Aleppo pine (*Pinus halepensis*), the maritime pine (*Pinus pinaster*) localized along the north‐eastern coast, and the Atlas cedar (*Cedrus atlantica*) fragmented on mountain tops across the country. The major aims were: (1) to identify the potential contact zone between the two mitochondrial clades, and the underlying genetic structure; (2) to understand if the ENA clade can be supported as an independent taxon, as hypothesized by Simonato et al. (2013), using both mitochondrial and nuclear markers that in previous works provided mostly congruent results (Santos et al. 2007; Simonato et al. 2007; Kerdelhué et al. 2015); and (3) to explore the main drivers of the genetic structure in this area, testing the role of host plant and possible influences of both past climate and recent anthropogenic changes.

## Materials and Methods

### Insect sampling

**Figure 2:**
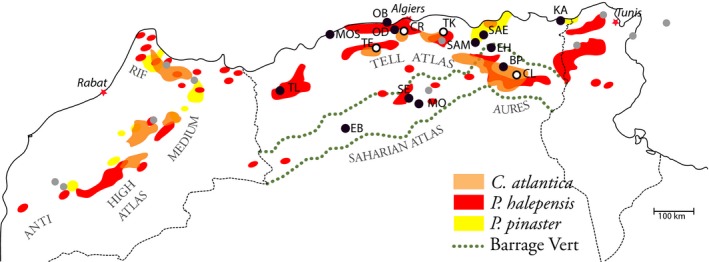
Map of north‐west Africa showing native distribution of pine (*Pinus halepensis* and *Pinus pinaster*) and Atlas cedar (*Cedrus atlantica*) with the indication of the macro‐scale sampling sites, country capital cities (red stars), and mountain chains (small capital letters). Host tree distribution is shown with colored spots: *P. halepensis* (red), *P. pinaster* (yellow), and *Cedrus atlantica* (orange). Host tree distributions were taken from Desmestrau et al. (1976), Vidakovic (1991) and Quezel (2000). Black circles indicating collection sites are filled on the basis of the host plant: black (*Pinus*) and white (*Cedrus*). In gray, the collection sites from Kerdelhué et al. (2009). The green dotted lines in southern Algeria indicate the approximate limit of the large afforestation project called “Barrage vert”, started in 1968 to stop desertification in the area. Aleppo pine was the most frequently tree species used in the project (Sahraoui 1995; Roques et al. 2015).

A total of 273 individuals of pine processionary moth, collected from July 2012 to August 2013 from 25 forest sites throughout Algeria, were used in this study (Table 1). Two rounds of samplings were performed, a macro‐scale sampling to cover all the range in Algeria, followed by a fine‐scale sampling focusing on the identified mitochondrial contact zone between the mitochondrial clades. In the macro‐scale sampling (Fig. 2), egg‐batches were collected at 16 locations (Table 1), covering three forest types where the pine processionary moth occurs, namely the low to medium elevation stands of Aleppo pine (*P. halepensis*; 11 sites), the high elevation stands of Atlas cedar (*C. atlantica*; four sites), and the coastal stands of maritime pine (*P. pinaster*; one site). Sites were chosen based on accessibility and no major areas where potential host‐plant species grow were excluded from the sampling (see distribution maps of the host plants and location of the sampling sites in Fig. 2). Special attention was paid to the area of the so‐called Barrage Vert, a belt of afforestation facing the Sahara desert established since 1968 to slow down the desertification process and to improve the local economy (Sahraoui 1995). Aleppo pine was the most frequent tree species used in the 120,000 ha of plantations, which may bridge the mountain forests in north‐eastern Algeria to the Saharian Atlas in the south, where moth has considerably increased in density (Zamoum and Démolin 2004; Zamoum et al. 2005). In order to reduce the risk of collecting siblings, each egg‐batch was collected from a different tree. Eggs were maintained at room temperature until hatching, after which ten first‐instar larvae were transferred to ethanol 70%. One larva per egg batch was further used in genetic analyses. Once the contact zone between mitochondrial clades was identified (see Results), a fine‐scale sampling was carried out to collect larvae from nine sites between OD and CR populations, all in stands of *P. halepensis*. Each larva was taken from a different tent, and each tent chosen from a different tree. Larvae were directly sampled in the field and immediately transferred to ethanol 70%. All ethanol‐preserved material was stored at −20°C. Information about sampling sites is given in Table 1.

**Table 1:** Sampling localities, ordered west to east, with the indication of the host‐plant species (*Ca, Cedrus atlantica; Ph, Pinus halepensis; Pp, Pinus pinaster*). Macro‐scale sampling localities are reported with codes in uppercase, whereas fine‐scale sampling localities are reported in lower case

Locality	Code	Geographical coordinates			Elevation (m)	Host plant
		Region (cardinal position)	Latitude	Longitude		
Tlémcen	TL	(SW)	34°36′48.96″N	1°01′50.56″W	1141	*Ph*
Mostaganem	MOS	(NW)	36°10′42.58″N	0°24′51.94″E	110	*Ph*
El bayedh	EB	(SW)	33°35′04.15″N	0°55′48.42″E	1385	*Ph*
Theniet El Had	TE	(NW)	35°51′19.64″N	2°00′07.33″E	1465	*Ca*
Oued El Belaa	OB	(SW)	36°36′56.82″N	2°14′24.72″E	59	*Ph*
Merad	*md*	(NW)	36°26′13.32″N	2°26′20.54″E	285	*Ph*
Es‐sahel	*es*	(NW)	36°27′19.78″N	2°30′05.26″E	327	*Ph*
Boumedfaa	*bm*	(NW)	36°23′28.88″N	2°31′14.79″E	209	*Ph*
Oued Djer	OD	(NW)	36°25′28.03″N	2°33′21.03″E	705	*Ph*
El Hachem	*elh*	(NW)	36°25′04.39″N	2°34′30.46″E	328	*Ph*
Ennhaoua	*en*	(NW)	36°23′49.99″N	2°38′02.34″E	262	*Ph*
Errayhane	*er*	(NW)	36°24′06.55″N	2°41′29.87″E	609	*Ph*
Sidi Madani	*sm*	(NW)	36°25′25.00″N	2°45′08.20″E	217	*Ph*
El Hamdania	*em*	(NW)	36°19′34.53″N	2°45′57.61″E	417	*Ph*
Bouarfa	*bf*	(NW)	36°27′04.42″N	2°49′30.43″E	605	*Ph*
Chréa	CR	(NW)	36°26′04.15″N	2°53′20.29″E	1453	*Ca*
Senalba	SE	(SW)	34°38′16.72″N	3°08′03.67″E	1306	*Ph*
Moudjbara	MO	(SW)	34°30′38.07″N	3°28′53.41″E	1055	*Ph*
Tikjda	TK	(NE)	36°26′58.54″N	4°07′27.64″E	1500	*Ca*
Setif Ain Messaoud	SAM	(NE)	36°12′10.97″N	5°16′03.04″E	1034	*Ph*
Setif Ain Kebira	SAE	(NE)	36°21′56.13″N	5°29′37.07″E	804	*Ph*
El Hassi	EH	(NE)	36°08′13.91″N	5°48′06.47″E	963	*Ph*
Batna	BP	(SE)	35°34′02.48″N	6°12′35.73″E	1203	*Ph*
Chélia	CL	(SE)	35°18′08.48″N	6°37′04.17″E	1933	*Ca*
El Kala	KA	(NE)	36°52′29.23″N	8°10′52.62″E	200	*Pp*

### Genetic analysis

DNA was extracted using a salting‐out procedure (Patwary et al. 1994). The number of individuals used for mitochondrial and microsatellite analyses is given in Table 2.

**Table 2:** Indices of genetic diversity in the populations analyzed for the mitochondrial and microsatellite datasets in the macro‐scale and fine‐scale (only microsatellite) sampling

Macro‐scale		mtDNA		Microsatellite					
Pop	*N*	Hd	*π*	*N*	Ae	Ra	He	Ho	%NA
TL	9	0.417 ± 0.191	0.0010 ± 0.0010	9	4.0	4.3	0.683	0.608	8.50
MOS	10	0.000 ± 0.000	0.0000 ± 0.0000	8	2.8	3.4	0.535	0.438	9.38
EB	8	0.464 ± 0.200	0.0008 ± 0.0008	10	3.8	4.2	0.661	0.650	6.27
TE	8	0.429 ± 0.169	0.0007 ± 0.0008	8	3.0	3.7	0.596	0.625	1.16
OB	10	0.200 ± 0.154	0.0003 ± 0.0005	10	2.9	3.7	0.643	0.620	3.95
OD	10	0.511 ± 0.164	0.0233 ± 0.0129	10	3.1	3.8	0.640	0.640	4.21
CR	9	0.583 ± 0.183	0.0010 ± 0.0010	9	2.9	3.6	0.571	0.510	4.29
SE	10	0.533 ± 0.180	0.0017 ± 0.0014	10	4.4	4.2	0.598	0.610	1.71
MO	6	0.533 ± 0.172	0.0008 ± 0.0009	10	3.1	3.7	0.594	0.550	7.19
TK	7	0.286 ± 0.196	0.0004 ± 0.0006	8	2.4	3.3	0.541	0.541	3.84
SAM	9	0.000 ± 0.000	0.0000 ± 0.0000	10	2.8	3.3	0.556	0.490	7.31
SAE	10	0.000 ± 0.000	0.0000 ± 0.0000	9	3.0	3.5	0.612	0.622	5.38
EH	10	0.000 ± 0.000	0.0000 ± 0.0000	9	3.5	3.9	0.631	0.600	6.25
BP	8	0.000 ± 0.000	0.0000 ± 0.0000	7	2.6	3.3	0.540	0.443	7.05
CL	8	0.464 ± 0.200	0.0008 ± 0.0008	9	3.1	3.8	0.605	0.607	1.41
KA	8	0.000 ± 0.000	0.0000 ± 0.0000	5	3.2	3.8	0.624	0.640	5.89

Fine‐scale	Microsatellite					
Pop	*N*	Ae	Ra	He	Ho	%NA
*md*	15	3.5	4.0	0.652	0.616	5.98
*es*	14	3.2	3.9	0.640	0.686	1.20
*bm*	13	3.3	3.9	0.659	0.687	3.47
*elh*	15	3.1	3.8	0.640	0.640	4.21
*en*	15	3.8	4.1	0.641	0.618	3.85
*er*	15	3.4	3.8	0.630	0.640	0.89
*sm*	15	3.9	4.1	0.659	0.680	3.09
*bf*	15	3.6	4.0	0.599	0.593	2.06
*em*	15	4.2	4.1	0.647	0.593	6.47

*N*, number of individuals; Hd, haplotype diversity; *π*, nucleotide diversity; He, expected heterozygosity; Ho, observed heterozigosity; Ra, average of rarefaction‐allelic richness; Ae, effective number of alleles per locus; %NA, percentage of null alleles.

For the macro‐scale sampling, a mitochondrial fragment of ca. 650 bp, corresponding to the contiguous genes tRNA‐Leu and Cytochrome Oxidase II (cox2), was amplified using the universal primers C1J2183 and TKN3772 (Simon et al. 2006). Polymerase chain reactions (PCR) were performed in final volumes of 20 μL containing 4 μL of 10 ×  PCR buffer (Promega, Madison, WI), 2 μL of 25 nmol/L MgCl_2_ (Promega), 1 μl of 2 mmol/L dNTPs (Promega), 0.5 U of Taq DNA polymerase (Promega), 1.0 μL of each primer (10 μmol/L), and 2 μL of template DNA. Cycling conditions started with an initial step of 96°C for 5 min followed by 35 cycles of 96°C for 1 min, 55°C for 1 min, 72°C for 1 min, and a final extension of 72°C for 5 min. PCR products were then sequenced with the primer TKN3772 at the BMR Genomics sequencing service (Padova, Italy). The sequences were aligned using MEGA version 6 (Tamura et al. 2013), and the final alignment was 648 bp long.

For the fine‐scale sampling, a clade‐specific PCR was developed and used in order to selectively amplify haplotypes belonging to one of the two mitochondrial clades found, that is the *pityocampa* or the ENA clade (Kerdelhué et al. 2009; and see Results). The primer C1J2183 was used together with alternatively one of the two clade‐specific primers designed using Primer3 (http://primer3.ut.ee/), namely hz‐tp (5′‐GAACATTGTCCATAGAAAG‐3′) and hz‐te (5′‐GGCTATTTAGTTCATCCAG‐3′), amplifying only the haplotypes belonging to either the *pityocampa* or the ENA clade, respectively (Figure S1).

The designed primer pairs amplified fragments of 1467 and 1155 bp for the *pityocampa* and ENA clade, respectively, encompassing a part of the Cytochrome Oxidase I (cox1) gene, the tRNA‐Leu and cox2. Each sample was amplified with both primer combinations. PCR conditions were the same as above except for the extension step, performed at 72°C for 1 min and 30 sec. PCR products were separated by gel electrophoresis using 1% agarose gel and visualized with SYBR Safe (Invitrogen, Carlsbad, CA). A subset of doubtful samples (12 individuals belonging to populations *md*,* bm*,* elh*,* en*,* er*,* sm,* and *em*) was sequenced in order to confirm the identity of the PCR bands, as amplifications were successful using both the *pityocampa* and the ENA‐specific primers.

For the microsatellite analysis, eleven loci were used to genotype the samples, namely Thpit7 – Thpit13 and Thpit15 – Thpit18, as described by Burban et al. (2012). Fluorescent PCR products were run and detected on an ABI 3730 automatic sequencer, and allele‐calling was performed using the Genemapper v4.0 software (Applied Biosystems, Foster City, CA). Two negative controls were used on each run to ensure that no contamination occurred. Genotyping was performed at the Genotyping and Sequencing facility of Bordeaux.

### Data analysis

#### mtDNA

Haplotype and nucleotide diversity in each population of the macro‐scale sampling was estimated by Arlequin version 3.5 (Excoffier and Lischer 2010). A haplotype parsimony network was reconstructed with all the haplotypes found using TCS 1.21 (Clement et al. 2000) as described by Templeton et al. (1992), with a probability cut‐off set at 95%. A phylogenetic tree was then built using a maximum likelihood (ML) method and the most general model of sequence evolution (GTR + I + G) using PhyML 3.0 software (Guindon et al. 2010) with neighbor‐joining starting trees and 100 bootstrap replicates. Three sequences, representing the *pityocampa* clade (origin: Moggio, Italy), the ENA clade (Bizerte, Tunisia) and the sister species *T. wilkinsoni*, were retrieved from GenBank (accession numbers HE963112, HE963113 and HE963116, Simonato et al. 2013) and used as references and outgroups.

The haplotypes found in this study were also compared with haplotypes found in the Iberian peninsula, Algeria, Morocco, Tunisia, and Libya obtained in a previous study (Kerdelhué et al. 2009) for part of the cox2 gene. A shorter region of 341 bp, corresponding to the overlapping fragment between these previous haplotypes and haplotypes from this present study, was then considered. This reduced data set and the cox2 haplotypes retrieved from Kerdelhué et al. (2009) were then used to build a ML phylogenetic tree using PhyML 3.0. Given the limited length of the fragment and the correlation between proportion of invariant sites and the parameter alpha of the gamma distribution (Nylander et al. 2004), we used the GTR model without considering the invariant + gamma parameters.

To assess the genetic structure of haplotypes among populations, a spatial analysis of molecular variance (SAMOVA 2.0, Dupanloup et al. 2002) was carried out on the whole dataset in order to identify groups of geographically homogeneous populations showing the highest differentiation among them. The F_CT_ coefficients corresponding to the proportion of total genetic variance due to differentiation between groups of populations were estimated for all values of *K* between 2 and 10 using a Kimura‐2 parameters model. The highest value of *F*_CT_ was then used to determine the best number of groups (Dupanloup et al. 2002). Within these groups, we tested past changes in demography through the Tajima's *D* and the Fu's *F*s tests (Tajima 1989; Fu and Li 1993). Mismatch distributions of the pairwise genetic differences (Rogers and Harpending 1992) were assessed testing a sudden expansion model through the sum of squared deviations between the observed and expected mismatch distributions obtained with a parametric bootstrap approach (1000 replicates). Both the neutrality tests and mismatch distribution analysis were calculated using Arlequin 3.5 (Excoffier and Lischer 2010).

#### Microsatellites

The ML of null alleles percentage (%NA) was estimated according to the EM algorithm (Dempster et al. 1977) using FreeNA (Chapuis and Estoup 2007). The expected (He) and the observed (Ho) heterozygosities, allelic richness (Ra, with a rarefaction at *n* = 5) and the effective number of alleles (Ae) for each population were calculated using Genetix 4.05.2 (Belkhir et al. 2004). Departure for Hardy–Weinberg equilibrium was not assessed given the low sampling size in almost all the populations. Pairwise *F*_st_ with ENA correction for the presence of null alleles was obtained using FreeNA (Chapuis and Estoup 2007), with a significance estimated by 1000 bootstrap replicates.

We assigned individuals to clusters based on their multilocus genotypes using a Bayesian inference method implemented in Structure 2.3.3 (Pritchard et al. 2000). The optimal number of clusters (*K*) represented by the data was determined with the Delta *K* method described in Evanno et al. (2005), implemented in Structure Harvester (Earl and vonHoldt 2012). As this method detects only the uppermost level of genetic structure (Evanno et al. 2005), the underlying layers of the genetic structure were detected using a hierarchical approach (Coulon et al. 2008). The admixture model with correlated allele frequencies and the locprior option was run for 20 replicates for each *K*, to check for Markov Chain Monte Carlo (MCMC) convergence, testing genetic groups from *K* = 1 to 10 for each level of the analysis. Each run consisted of a burn‐in period of 20,000 MCMC steps followed by 80,000 iterations. The method was repeated until each cluster could not be divided further. For this reason, the posterior probability of the data [ln*P*(*D*)] for each value of *K* was checked to determine if the ln*P*(*D*) value was maximum for *K* = 1. As in Coulon et al. (2008), individuals with maximum inferred ancestry <0.6 were not assigned to any group and considered as exhibiting admixed assignation. Graphs with the best ln*P*(*D*) for a given *K* were chosen and displayed using DISTRUCT 1.1 (Rosenberg 2004).

Finally, we carried out an analysis of the molecular variance (AMOVA, Excoffier et al. 1992) on the microsatellite dataset considering only the macro‐scale sampling in order to assess the percentage of variance explained by the mitochondrial clades (considering both the *pityocampa* and ENA clades and the SAMOVA population assignments), and by the host plant (grouping populations collected on cedar or pine). AMOVA was performed with Arlequin using *F*_st_ as genetic distances with 10,000 permutations per run.

## Results

### mtDNA

A total of 24 haplotypes was obtained from the alignment of the 648‐bp‐long fragment sequenced for 140 individuals. Several populations showed low values of gene diversity (Hd) mainly in the east part of Algeria, whereas the highest values were found in populations both in the northern part (OD and CR) and in the south‐western part of Algeria (SE and MO) (Table 2). The highest values of nucleotide diversity (*π*) were found in the northern population of OD (*π* = 0.0233) and in the south‐western population of SE (*π* = 0.0017). In the south‐eastern region of the country, population CL was the only one showing a *π* value different from zero (Table 2).

**Figure 3:**
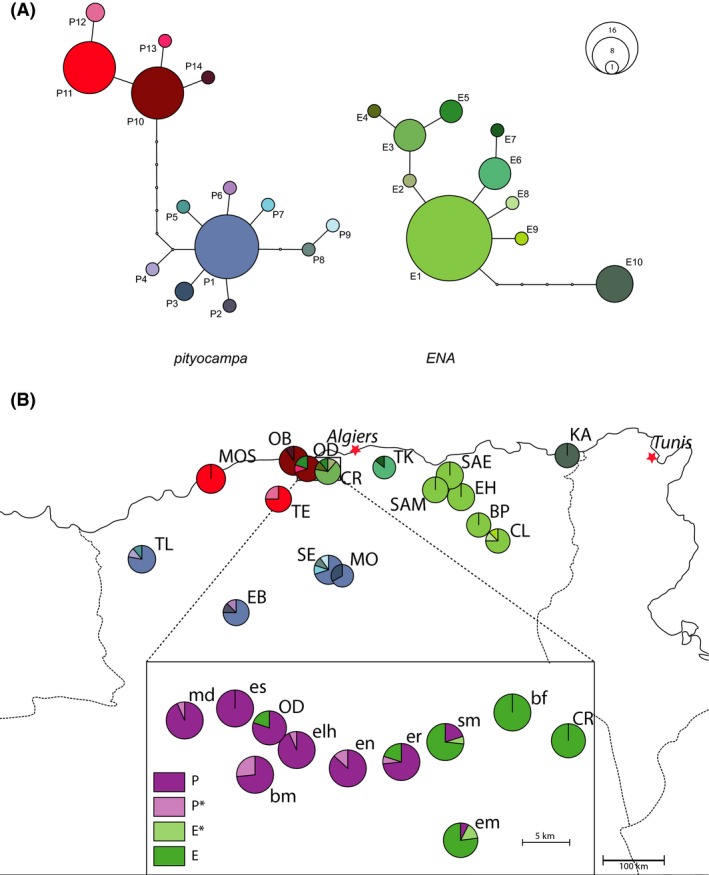
(A) TCS network showing phylogenetic relationships among haplotypes of the tRNA‐Leu‐cox2 fragment 648 bp long. P: *pityocampa* clade, E: ENA clade. (B) Geographic distribution of haplotypes of *pityocampa* and ENA clades, with the populations from the contact zone reported in the inset. Population codes are given in Table 1. P*: individuals positive for both *pityocampa*‐ and ENA‐specific PCR that resulted to belong to *pityocampa* clade after sequencing; E*: individuals positive for both *pityocampa*‐ and ENA‐specific PCR that resulted to belong to ENA clade after sequencing.

The haplotype network (Fig. 3A) and phylogenetic tree (Figure S2) showed that the haplotypes found were related to both clades found in Kerdelhué et al. (2009), with 14 haplotypes belonging to the *pityocampa* clade (P1–P14) and 10 haplotypes (E1–E10) belonging to the ENA clade. The two clades showed a disjoint geographical distribution, with the *pityocampa* haplotypes found in the western part of the country, whereas the ENA haplotypes were found only in the east (Fig. 3B). A very localized contact zone between the two clades was identified in the north around population OD in the Tell Atlas, the only site showing mitochondrial haplotypes from both clades. All the other populations exclusively contained haplotypes belonging either to the *pityocampa* or the ENA clade.

Using the complete alignment of the 24 Algerian haplotypes, the network showed that the *pityocampa* clade was divided into two main sub‐clades. The first one grouped most of the haplotypes found in the north‐western part of Algeria (OB, OD, MOS and TE). It contained two main haplotypes (P10 and P11) and three rare ones diverging from the two main haplotypes by just one mutational step. The other sub‐clade corresponded to the south‐western populations TL, EB, SE, and MO. It showed a star‐shaped structure, with one frequent haplotype (P1) and 8 low‐frequency haplotypes differing by a few (1–3) mutational steps. The two *pityocampa* sub‐clades were separated by seven mutational steps and were found in different regions (i.e., no site contained individuals from both sub‐clades) (Fig. 3). The ENA clade also showed a mostly star‐shaped structure, with one major haplotype (E1) and nine rare ones shared by 1–8 individuals. Interestingly, one of these haplotypes (E10) was differentiated from the other ENA haplotypes by five mutation steps and found in all individuals sampled in the easternmost site KA. Finally, in both the *pityocampa* and ENA clades the low‐frequency haplotypes were mainly population’ specific haplotypes (Fig. 3B).

**Figure 4:**
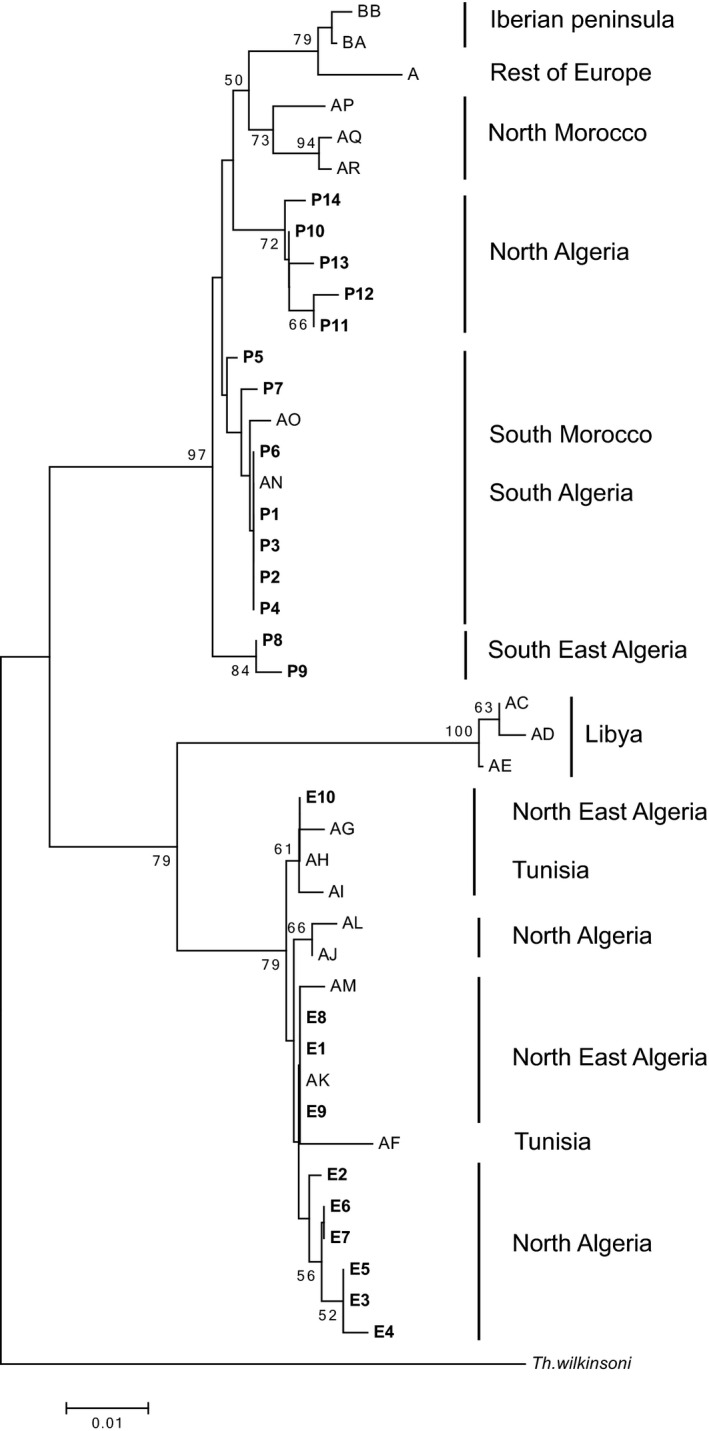
Maximum likelihood consensus tree of the fragment of 341 bp matching the cox2 haplotypes from Algeria (present study, reported in bold) with those from N Africa, Iberian Peninsula and rest of Europe found in Kerdelhué et al. (2009). *Thaumetopoea wilkinsoni* from the Middle East has been taken as an outgroup from the same paper.

After reducing the sequence data to the 341 bp also used in Kerdelhué et al. (2009), the phylogenetic tree showed that the *pityocampa* haplotypes found in North Algeria were distinct but closely related to the haplotypes previously found in North Morocco, while most haplotypes found in southern Algeria in this present study were similar to haplotypes previously found in the southern part of Morocco (Fig. 4). Interestingly, haplotypes P8 and P9 were related to this clade but separated by two mutation steps. For the ENA clade, the haplotypes found are not clustered in well‐supported sub‐clades. Almost all the haplotypes are closely related to haplotypes previously found in North Algeria, with the exception of E10, the haplotype found in KA, that is linked to three of the four Tunisian cox2 haplotypes found in Kerdelhué et al. (2009).

The SAMOVA algorithm (Dupanloup et al. 2002) was used to determine the number of geographically homogeneous groups maximally differentiated from each other inside the whole mtDNA dataset. The number of groups producing the maximum values of *F*_CT_, that is the proportion of total genetic variance due to differentiation between groups of populations, was eight (*K* = 8) and retained as the best option to group populations (Table S1). For each of these homogeneous groups, Tajima's *D* and Fu's *F*s were estimated and tested in order to check for past demographic events (Table 3). Both tests were significantly negative for the EB‐MO‐SE‐TL group in the *pityocampa* clade and for the CR and the SAM‐SAE‐EH‐BP‐CL groups in the ENA clade. Mismatch analyses were consistent with the sudden expansion model for all the SAMOVA groups except for OD and KA (Table 3).

**Table 3:** Results of the neutrality tests (Tajima's *D*, Fu's *F*s) and mismatch distribution analysis applied on the SAMOVA population groups obtained for *K* = 8

Clade	SAMOVA groups	*D*	*F*s	SSD
*pityocampa*	MOS‐TE	−0.53	−0.01	0.32077
	EB‐MO‐SE‐TL	−2.06**	−6.48**	0.00044
	OB	−1.11	−0.34	0.33101
ENA	CR	−1.51	−1.89*	0.02385
	TK	−1.00	−0.09	0.24889
	SAM‐SAE‐EH‐BP‐CL	−1.48*	−2.89**	0.00007
	KA	0.00	–	0.00000**

SSD, sum of squared deviations

OD excluded due to the mixed origin of individuals

*P < 0.05, **P < 0.01.

Based on clade specific primers amplifying a fragment of cox1‐tRNALeu‐cox2 genes, individuals along the 40 km transect between OD and CR were assigned either to the *pityocampa* or the ENA clade. The 12 individuals amplified by both clade‐specific primers were clearly assigned to a single clade after sequencing. PCR results showed that western populations (*md*,* es*,* bm*,* elh*,* en*) belonged to the *pityocampa* clade, central populations (*er*,* sm*,* em*) were composed of individuals belonging to both clades, while the easternmost population (*bf*) belonged to the ENA clade.

### Microsatellites

#### Standard diversity indices and heterozygosity

Two loci (Thapit09 and Thapit17) showed a high frequency of null alleles in all the populations analyzed (Table S2) and they were therefore excluded from the subsequent analyses. The other nine loci showed a percentage of null alleles across populations between 0.68% and 5.65%.

The average number of alleles per locus was found to be quite similar among sites, ranging between 3.3 and 4.2 (Table 2). Pairwise *F*_st_ obtained with the ENA correction for the presence of null alleles (Chapuis and Estoup 2007), reported in Table 4, showed significant levels of differentiation mainly between north‐western populations (MOS, TE, OB, OD, CR, and TK) and eastern populations (SAM, SAE, EH, BP, CL, and KA) with *F*_st_ values ranging from 0.137 to 0.231, and between population KA and all the other sites (*F*_st_ values between 0.127 and 0.202).

**Table 4:** Pairwise *F*_st_ matrix obtained using nine microsatellite loci after applying the ENA correction for null alleles using FREENA; (a) macro‐scale sampling population matrix; (b) fine‐scale sampling population matrix

	TL	MOS	EB	TE	OB	OD	CR	SE	MO	TK	SAM	SAE	EH	BP	CL
a)															
MOS	**0.121**														
EB	**0.027**	**0.177**													
TE	**0.107**	**0.057**	**0.156**												
OB	**0.079**	**0.076**	**0.136**	**0.055**											
OD	**0.063**	**0.099**	**0.076**	**0.082**	**0.054**										
CR	**0.156**	**0.122**	**0.183**	**0.032**	**0.111**	**0.127**									
SE	**0.065**	**0.123**	**0.028**	**0.144**	**0.124**	**0.065**	**0.179**								
MO	**0.050**	**0.136**	**0.045**	**0.153**	**0.129**	**0.080**	**0.199**	**0.002**							
TK	**0.150**	**0.088**	**0.193**	**0.060**	**0.133**	**0.135**	**0.050**	**0.176**	**0.192**						
SAM	**0.137**	**0.172**	**0.098**	**0.188**	**0.195**	**0.171**	**0.231**	**0.070**	**0.089**	**0.186**					
SAE	**0.123**	**0.173**	**0.110**	**0.188**	**0.171**	**0.173**	**0.208**	**0.100**	**0.110**	**0.153**	**0.023**				
EH	**0.106**	**0.164**	**0.068**	**0.178**	**0.176**	**0.139**	**0.193**	**0.047**	**0.058**	**0.157**	**0.022**	**0.026**			
BP	**0.108**	**0.173**	**0.083**	**0.187**	**0.167**	**0.137**	**0.213**	**0.055**	**0.052**	**0.186**	**0.024**	**0.055**	**0.015**		
CL	**0.099**	**0.161**	**0.094**	**0.168**	**0.160**	**0.149**	**0.197**	**0.072**	**0.057**	**0.173**	**0.035**	**0.034**	**0.028**	−0.021	
KA	**0.162**	**0.179**	**0.163**	**0.186**	**0.202**	**0.167**	**0.175**	**0.140**	**0.178**	**0.159**	**0.182**	**0.161**	**0.127**	**0.159**	**0.152**

	*md*	*es*	*bm*	*elh*	*en*	*er*	*sm*	*em*
b)								
*es*	−0.008							
*bm*	−0.003	**0.004**						
*elh*	**0.009**	−0.003	**0.001**					
*en*	**0.019**	**0.007**	**0.012**	**0.015**				
*er*	**0.018**	**0.022**	**0.009**	**0.020**	**0.030**			
*sm*	−0.003	−0.002	**0.002**	**0.002**	**0.005**	−0.001		
*em*	**0.032**	**0.036**	**0.023**	**0.029**	**0.024**	**0.005**	**0.007**	
*bf*	**0.020**	**0.031**	**0.019**	**0.037**	**0.030**	**0.012**	**0.005**	**0.020**

Estimates for which the 95% confidence intervals after 1000 bootstraps did not include zero are reported in bold.

#### Population genetic structure

**Figure 5:**
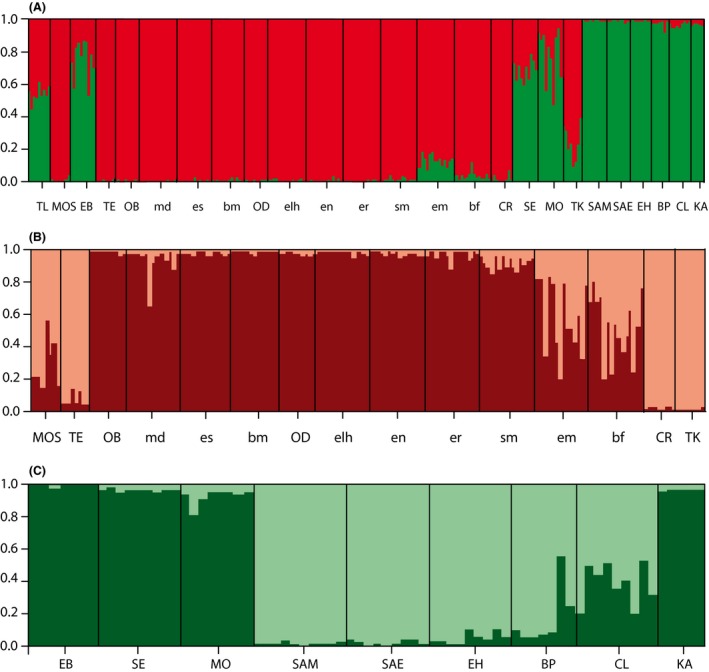
Bar plot indicating the probability of microsatellite assignment based on the Structure analysis grouped by population. (A) Whole dataset for *K* = 2. (B) Populations from north‐western Algeria for *K* = 2. (C) Populations from southern and eastern Algeria for *K* = 2.

The genetic structure among the sampled populations was assessed with the Bayesian clustering analysis implemented in Structure. Results were consistent among the 20 runs of each selected *K*. Four distinct groups distributed across two hierarchical scales were identified. At the uppermost level of population structure, individuals were assigned to two main groups (Fig. 5A). One group contained all individuals collected in the north‐western part of Algeria (belonging to populations MOS, TE, OB, OD, CR, TK, and to populations collected along the transect) with individuals from population TK showing a low admixture level (<40%) with the second group. The second group clusters all individuals from the eastern part of Algeria (populations SAM, SAE, EH, BP, CL, KA) together with all individuals from the southern part of Algeria (populations EB, SE, MO), which are partially admixed with the first group. Almost all individuals (except one, assigned to the second group) from TL population showed a mixed assignment (none of them had a *q*‐value over 60% for a group). Population TL was thus not included in the following step. The second and final round of the hierarchical analysis divided each group into two further sub‐groups. Individuals belonging to the north‐western group were split between a sub‐group including populations OB, OD, and all the transect populations, and another sub‐group including populations TE, CR, TK, and MOS (Fig. 5B). Individuals from populations *em* and *bf* in the contact zone and, to a lesser extent, individuals from population MOS exhibited a mixed assignment between the two sub‐groups. The second group was split between a sub‐group comprising the populations from the south (EB, SE, MO) and the population KA from the easternmost part of Algeria, and another sub‐group comprising individuals from populations SAM, SAE, EH, BP, and CL, with individuals from BP and CL showing a mixed assignment with the first sub‐group (Fig. 5C).

The AMOVA analysis showed a low but still significant percentage of variance (2.97% *P* < 0.05) explained by the main mitochondrial clades (*pityocampa* and ENA) that significantly increased when considering the clustering based on the SAMOVA population assignment (10.82%, *P* < 0.001). When considering groupings based on host plant the percentage of variance explained was again low although significant (3.5%, *P* < 0.05) (Table 5).

**Table 5:** Results of AMOVA tests on the microsatellite dataset divided according to the SAMOVA mitochondrial groups for *K* = 2 and *K* = 8 and host plant (pine and cedar)

	Variance components	Percentage of variation
Whole dataset		
Among pops	0.391 Va	12.21***
Within pops	2.812 Vb	87.79***
mtDNA: SAMOVA groups for *K* = 2 (*pityocampa*/ENA clades)		
Among groups	0.097 Va	2.97*
Among pops within groups	0.345 Vb	10.61***
Within pops	2.813 Vc	86.43***
mtDNA: SAMOVA groups for *K* = 8		
Among groups	0.352 Va	10.82***
Among pops within groups	0.092 Vb	2.82***
Within pops	2.812 Vc	86.36***
Host plant: Cedar(CR,TE,TK,CL)/Pine(TL,MOS,EB,OB,OD,SE,MO,SAM,SAE,EH,BP,KA)		
Among groups	0.115 Va	3.50*
Among pops within groups	0.346 Vb	10.58***
Within pops	2.812 Vc	85.92***

*P < 0.05, ***P < 0.001

## Discussion

We here extended the population genetic analysis of the pine processionary moth in Algeria, previously outlined in a broader phylogeographic study covering the whole range (Kerdelhué et al. 2009) that considered only two remote sites in this area. Moreover, we considered both mitochondrial and microsatellite markers for several populations collected in almost all the species range in the country. Phylogenetic analyses better outlined the contact zone between the two main mitochondrial clades identified in this species, the *pityocampa* and the ENA clade, showing a distinct geographical distribution with a narrow overlapping area. Interestingly, the genetic pattern found with microsatellite markers does not fully mirror the mitochondrial differentiation, raising questions about the taxonomical status of the two clades.

### mtDNA

The allopatric distribution of the two mitochondrial clades is consistent with a secondary contact after a range expansion from refugial populations, probably located in the mountains of southern Morocco (High and Medium Atlas) and Algeria (Saharian Atlas) for the *pityocampa* clade, and in the Tunisian mountains for the ENA clade, as can be inferred from the phylogenetic tree that includes the haplotypes from Kerdelhué et al. (2009). The mitochondrial contact zone between the *pityocampa* and the ENA clades, ranging from Oran on the Mediterranean coast to the Saharian Atlas in the south, shows almost no overlap between the two clades, as shown by the analysis of the population along the 40 km transect where mixed populations composed by individuals belonging to both clades were limited to a quite narrow area. Interestingly, other species of low dispersing animals show a genetic differentiation between western and eastern Maghreb, dating back in a range between 0.018 and 15 million year ago, explained by several geological and climatic events (Guiller and Madec 2010). Concerning the split between the *pityocampa* and ENA clades, as moth distribution is usually conditioned by both climate and geographical barriers, a conjunction between geological events (the rise of the Tellian Atlas) and the late Tertiary climatic change has been suggested as a possible explanation (Kerdelhué et al. 2009).

Further major biogeographical events probably shaped the differentiation between the north‐western and the south‐eastern sub‐clades within *pityocampa* clade in Algeria (Fig. 3), leading to a further host plant range fragmentation and thus to the isolation of moth populations on mountain tops in both Saharian and Tell Atlas. Interestingly, the phylogenetic tree including all the Maghreb sites showed that the *pityocampa* haplotypes found in the south of Algeria are clustered together with those found in southern Morocco, suggesting a past connection of the populations between the Saharan and the High and Middle Atlas. Conversely, populations along the Moroccan and Algerian coast belong to two distinct and supported sub‐clades, indicating a past fragmentation of the moth range.

In eastern Maghreb, haplotypes belonging to the ENA clade do not show clear and well‐supported sub‐clades, although eastern Algerian haplotypes seem to be more closely associated with the Tunisian ones. This pattern could be due to a more continuous host plant distribution in eastern Maghreb and to the lack of barriers affecting the genetic structure of this clade. The gap observed in the network (Fig. 3A) between the haplotype in the easternmost population KA and the other Algerian ENA haplotypes may be ascribed to missing sampling sites rather than to past gaps of the range. Yet, only a fraction of the ENA range was sampled here, and geographical limits of the sub‐clades found in Kerdelhué et al. (2009) still need to be clarified.

Climatic oscillations and host plant range shifts at the end of Pleistocene could have influenced both the *pityocampa* and the ENA clade populations, given the star‐shaped structure found in both clades and the negative values of the neutrality test for the southern populations, suggesting past bottlenecks followed by rapid expansions. Mountain ranges could have served as refugia during the warmer periods, as found for moderate elevation populations of western Europe (Rousselet et al. 2010), with moth populations tracking their optimal environment by movements along mountain slopes rather than south–north dispersal, as observed between Morocco and Europe through the Gibraltar strait (Kerdelhué et al. 2009). Conversely, the expansion of southern populations of both clades could be due to the recent afforestation program of Barrage Vert along the Saharian Atlas (Sahraoui 1995; Fig. 2).

Finally, host plant range fragmentation could have influenced the genetic structure, as may be suggested by SAMOVA results, although more precise data about past host plant distribution would be needed to confirm this hypothesis. The recent finding of high population densities on *C. atlantica* in the Algerian mountains (Sbabdji and Kadik 2011) is likely the results of a shift to high elevation from the lower pine forests, although it may indicate that this conifer is highly suitable to the pine processionary moth and could have been used in the past in high‐elevation refugial areas, in the same manner that mountain pines played a role in the recent history of the species in Europe (Rousselet et al. 2010).

### Microsatellites

The microsatellite analyses, carried out with a Bayesian individual assignment, showed a pattern of genetic variability similar to that obtained with mitochondrial markers, although less pronounced. North‐western (MOS, TE, OB, OD, CR, and TK and all contact zone populations) and eastern (SAM, SAE, EH, BP, CL, KA) populations seem to cluster in two genetically homogeneous groups. The subdivision between the north‐western and the eastern groups contrasts with the less clear differentiation in the south‐west (TL, EB, SE, MO), showing admixed assignments in the Structure analysis. The hierarchical analysis within each group showed a further genetic structure. In the north‐western group, populations along the coast (OB, OD, and all the transect populations) are separated from those at higher elevations (TE, CR, TK), although MOS, along the coast, joins the latter. This pattern could suggest a prime role of elevation or host plant in population differentiation, possibly through assortative mating. The relative effect of elevation or association to cedar cannot be disentangled as both factors are tightly linked, although a low but still significant association of the genetic variance to host plant was found. A more complex pattern has been found in the eastern group that also shows a closer similarity to the south‐western populations. These latter populations (EB, SE, MO) together with the easternmost population KA form a distinct cluster, differentiated from the north‐eastern populations (SAM, SAE, EH, and BP). This pattern could be due to a partial connection of the host plant range between the Saharan Atlas and the eastern mountains (Aurès) in the south, possibly favored by the recent, large afforestation efforts in the Barrage Vert bridging islands of native stands (Sahraoui 1995; Fig. 2). This hypothesis could also explain the high frequency of individuals exhibiting admixed assignments in the westernmost population TL, which may be the result of a recent contact between north‐western and south‐western groups in an area previously genetically isolated. Nonetheless, although estimated from a sub‐optimal number of individuals per population, the highest values of pairwise genetic distances among the north‐western, the south‐western, and the north‐eastern populations (*F*_st_ = 0.186 between TK and SAM or BP, *F*_st_ =0.199 between CR and SE, and *F*_st_ = 0.231 between CR and SAM) show that these sub‐groups are still well differentiated, with *F*_st_ values similar to those observed between geographically distant (Kerdelhué et al. 2006) or phenologically different (Santos et al. 2007) populations in Europe. This differentiation could be due to the presence of geographical barriers, or to host fragmentation impeding migration and gene flow.

### Comparison between mtDNA and microsatellites

The comparison of the main genetic groups identified with the nuclear microsatellite data and the *pityocampa*/ENA mitochondrial clades underlines the lack of a strict concordance between mitochondrial and nuclear markers. This holds true both at higher geographical scale and along the transect corresponding to the mitochondrial contact zone. A conflicting pattern between mitochondrial and nuclear markers, found also in several other animal species, is usually ascribed to causes ranging from adaptive introgression to demographic disparities and sex‐biased asymmetric gene flow (Toews and Brelsford 2012). In this study, the discordance could be the result of a sex‐biased dispersal, with males spreading longer distances than females as shown in other populations for both *T. pityocampa* and *T. wilkinsoni* (Salvato et al. 2002, 2005; Simonato et al. 2007), leading to a nuclear gene flow higher than the mitochondrial one. Moreover, the introgression for organelle DNA whenever associated with the least dispersing sex, is expected to be highly asymmetrical and usually goes from the local to the invading taxon (Currat et al. 2008; Petit and Excoffier 2009). In our case, a slight asymmetry has been detected in the northern populations CR and TK, which belong to the ENA clade when taking into account the mitochondrial DNA, whereas they are grouped with north‐western populations when the nuclear markers are considered. This pattern may suggest an eastward shift of the north‐western group into the eastern one. An opposite asymmetry, with individuals with a *pityocampa* mitochondrial DNA showing nuclear markers rather associated to eastern populations, has been observed in the south‐western populations. This pattern contrasts with that observed in the north and may result from demographic asymmetries (i.e., different population size) (Toews and Brelsford 2012), which could have been enhanced by anthropogenic changes (the recent afforestation of Barrage Vert).

The discrepancy between the two markers is lower when comparing the nuclear genetic groups with the genetic structure found inside the mitochondrial clades, as shown by the higher percentage of nuclear genetic variance explained by the mtDNA subgroups. This suggests a possible influence of habitat fragmentation on the nuclear gene flow among moth populations in this area.

## Conclusions

The comparison of the mitochondrial and nuclear markers underlines the strong geographical structure of the pine processionary moth populations at the southern edge of its range, as a result of the particular topography of the area, which has affected the past and current distribution of the host plants. The lack of a strict overlap between the two kinds of markers highlights an ongoing introgression between the *pityocampa* and the ENA clades, excluding the presence of strong genetic barriers, and thus suggesting that the two clades could not be considered as distinct taxa. Nonetheless, a thorough analysis of morphological traits of local populations would be required to further confirm the taxonomical status of these two clades. Finally, the role of the large afforestation effort in the “Barrage Vert” belt should be better explored in order to understand how much human activity might interfere with gene flow among isolated populations.

## Conflict of Interest

None declared.

## Supporting information


**Figure S1.** Clade‐specific primer map.
**Figure S2.** Maximum likelihood consensus tree of the tRNALeu‐cox2 haplotypes found in this study (648 bp long, P and E stand for *pityocampa* and ENA clades, respectively).

**Table S1.** F*ct* values for the different number of groups (K) of population inferred by SAMOVA algorithm on the whole mtDNA dataset.
**Table S2.** Total number of alleles per locus and average null alleles percentage (%NA) per locus.
