# Supplementary material for: Genetic differentiation of the pine processionary moth at the southern edge of its range: contrasting patterns between mitochondrial and nuclear markers
Source: Ecol Evol. 2016 May 26;6(13):4274–88. doi: 10.1002/ece3.2194 (PMC4884675; doi:10.1002/ece3.2194)
Supplement: Supplementary file 1 — Figure S1. Clade‐specific primer map. Figure S2. Maximum likelihood consensus tree of the tRNALeu‐cox2 haplotypes found in this study (648 bp long, P and E stand for pityocampa and ENA clades, respectively). [file ECE3-6-4274-s001.docx]

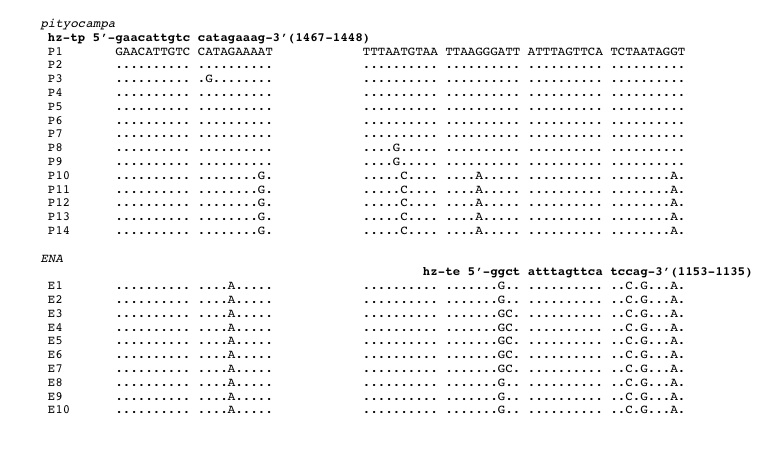


Fig. S1. Clade-specific primer map.


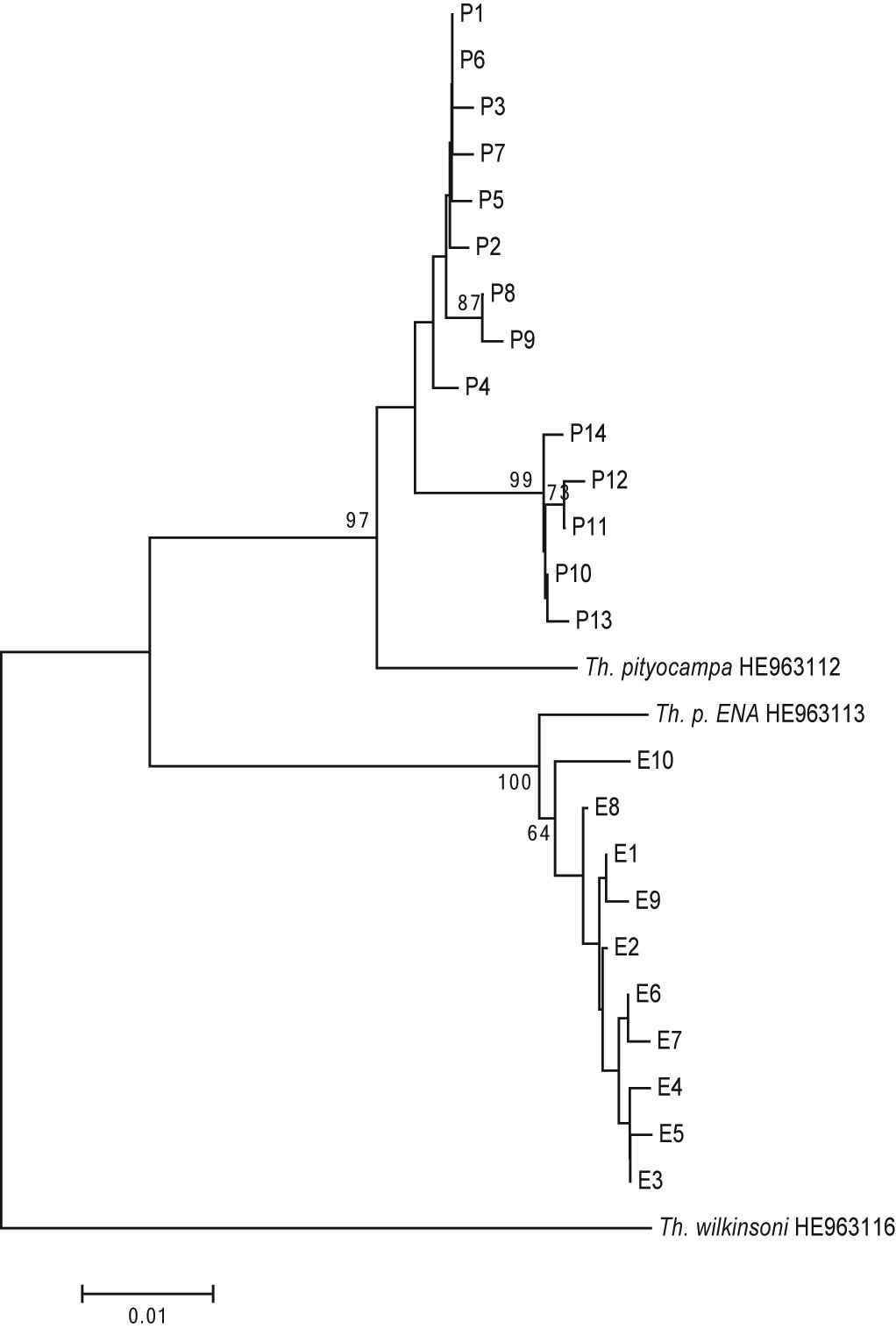


**Figure S2.** Maximum likelihood consensus tree of the tRNALeu-cox2 haplotypes found in this study (648 bp long, P and E stand for *pityocampa* and ENA clades, respectively). Three sequences belonging to *T. pityocampa*, *T. pityocampa* ENA and *T. wilkinsoni* were added from Simonato *et al.* 2013.
