# Supplementary material for: Genetic differentiation of the pine processionary moth at the southern edge of its range: contrasting patterns between mitochondrial and nuclear markers
Source: Ecol Evol. 2016 May 26;6(13):4274–88. doi: 10.1002/ece3.2194 (PMC4884675; doi:10.1002/ece3.2194)
Supplement: Supplementary file 2 — Table S1. Fct values for the different number of groups (K) of population inferred by SAMOVA algorithm on the whole mtDNA dataset. Table S2. Total number of alleles per locus and average null alleles percentage (%NA) per locus. [file ECE3-6-4274-s002.doc]

**Table S1.** F*ct* values for the different number of groups (K) of population inferred by SAMOVA algorithm on the whole mtDNA dataset. Population groups belonging to the *pityocampa* clade are reported on the left of the group composition column, while the ENA population groups are reported on the right. In bold the population with individuals belonging to both clades (OD).

| K | Group composition  *pityocampa* ENA | Fct |
| --- | --- | --- |
| 2 | (TL,MOS,OB,**OD**,EB,SE,MO,TE) (TK,CR,BP,CL,EH,SAE,SAM,KA) | 0.879* |
| 3 | (MOS,OB,**OD**,TE)(TL,EB,SE,MO) (TK,CR,BP,CL,EH,SAE,SAM,KA) | 0.921* |
| 4 | (MOS,OB,**OD**,TE)(TL,EB,SE,MO) (TK,CR,BP,CL,EH,SAE,SAM)(KA) | 0.934* |
| 5 | (MOS,OB,TE)(TL,EB,SE,MO)(**OD**) (TK,CR,BP,CL,EH,SAE,SAM)(KA) | 0.940* |
| 6 | (MOS,OB,TE)(TL,EB,SE,MO)(**OD**) (CR)(TK,BP,CL,EH,SAE,SAM)(KA) | 0.944* |
| 7 | (MOS,TE)(OB)(TL,EB,SE,MO)(**OD**) (CR,TK)(BP,CL,EH,SAE,SAM)(KA) | 0.947* |
| 8 | (MOS,TE)(OB)(TL,EB,SE,MO)(**OD**) (CR)(TK)(BP,CL,EH,SAE,SAM)(KA) | 0.951* |
| 9 | (MOS,TE)(OB)(TL,EB)(SE)(MO)(**OD**) (CR,TK)(BP,CL,EH,SAE,SAM)(KA) | 0.945* |
| 10 | (MOS,TE,OB)(TL,EB,SE)(MO)(**OD**) (CR)(TK)(BP)(CL)(EH,SAE,SAM)(KA) | 0.939* |

**Table S2.** Total number of alleles per locus and average null alleles percentage (*%NA*) per locus.

| Locus | Total number of alleles | *%NA* |
| --- | --- | --- |
| Thpit7 | 18 | 1.61 |
| Thpit8 | 21 | 2.29 |
| Thpit9 | 17 | 24.65 |
| Thpit10 | 10 | 3.56 |
| Thpit11 | 8 | 5.65 |
| Thpit12 | 5 | 3.10 |
| Thpit13 | 37 | 1.65 |
| Thpit15 | 40 | 0.68 |
| Thpit16 | 21 | 1.57 |
| Thpit17 | 3 | 19.36 |
| Thpit18 | 6 | 4.12 |
